# The radiology job market in the Netherlands: which subspecialties and other skills are in demand?

**DOI:** 10.1007/s00330-023-09983-5

**Published:** 2023-08-11

**Authors:** Ton Velleman, Walter Noordzij, Rudi A.J.O. Dierckx, Thomas C. Kwee

**Affiliations:** 1 Medical Imaging Center, Departments of Radiology & Nuclear Medicine and Molecular Imaging, University Medical Center Groningen, Groningen, the Netherlands; 2 Department of Radiology, Nuclear Medicine and Molecular Imaging, University Medical Center Groningen, University of Groningen, Hanzeplein 1, P.O. Box 30.001, 9700 RB Groningen, the Netherlands

**Keywords:** Employment, Radiologist, Nuclear medicine, Residency, Education

## Abstract

**Abstract:**

**Objectives:**

To evaluate the current job market for medical specialists in radiology and nuclear medicine (NM) in the Netherlands.

**Methods:**

Vacancies posted for radiologists and nuclear medicine physicians in the Netherlands between December 2020 and February 2022 were collected and analyzed.

**Results:**

A total of 157 vacancies (146 for radiologist and 11 for nuclear medicine physicians) were included. The most sought-after subspecialties were all-round (22%), abdominal (19%), and interventional radiology (14%), and 30% of vacancies preferred applicants with additional non-clinical skills (research, teaching, management, information and communications technology (ICT)/artificial intelligence (AI)). Non-academic hospitals significantly more frequently requested all-round radiologists (*n *= 31) than academic hospitals (*n *= 1) (*p *= 0.001), while the distribution of other requested subspecialties was not significantly different between non-academic and academic vacancies. Non-academic hospitals also significantly more frequently requested additional research tasks in their vacancies (*n *= 35) compared to academic hospitals (*n *= 4) (*p *= 0.011). There were non-significant trends for non-academic hospitals more frequently requesting teaching tasks in their vacancies (*n *=18) than academic hospitals (*n *= 1) (*p *= 0.051), and for non-academic hospitals more frequently asking for management skills (*n *= 11) than academic hospitals (*n *= 0) (*p *= 0.075).

**Conclusion:**

All-round, abdominal, and interventional radiologists are most in demand on the job market in the Netherlands. All-round radiologists are particularly sought after by non-academic hospitals, whereas nuclear radiologists who completed the Dutch integrated NM and radiology residency seem to be welcomed by hospitals searching for a nuclear medicine specialist. Finally, non-clinical skills (research, teaching, management, ICT/AI) are commonly requested. These data can be useful for residents and developers of training curricula.

**Clinical relevance statement:**

An overview of the radiology job market and the requested skills is important for residents, for those who seek work as a radiologist, and for those who are involved in the design and revision of residency programs.

**Key Points:**

- *Review of job vacancies over an extended period of time provides valuable information to residents and feedback to potentially improve radiology and nuclear medicine (NM) residency programs.*
- *All-round radiologists are wanted in non-academic hospitals and nuclear radiologists (those who have completed an integrated NM-radiology curriculum) are welcomed by hospitals searching for nuclear medicine specialists in the Netherlands.*
- *There is a need to train residents in important non-clinical skills, such as research and teaching, but also management and communications technology/artificial intelligence.*

## Introduction

Job opportunities for radiologists and nuclear medicine physicians continue to be a point of concern and discussion in Europe and the USA [1, 2]. The general perception is that the job market is overflooded and that vacancies are scarce [1, 2]. Factors that bring additional uncertainty for the job market in Europe and the USA are ongoing cuts in healthcare reimbursement, the continuing increase in workload for radiologists, and uncertainties regarding the effect of developments in artificial intelligence (AI) on daily practice [3–7]. Furthermore, with the increased focus on inclusion and diversity, the number of female radiologists is expected to increase [8]. Female radiologists more often work part-time than male radiologists, which might lead to an even higher demand for radiologists in the future [8].

**Fig. 1:**
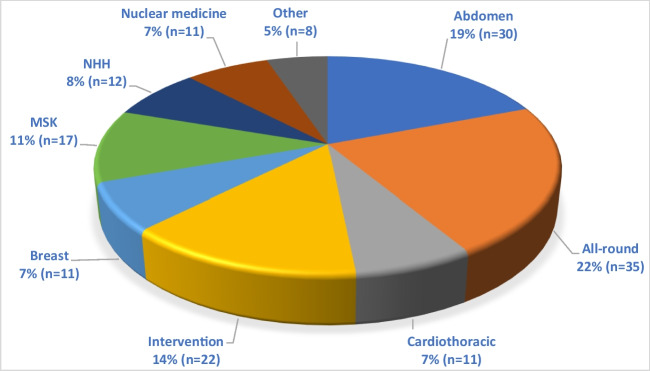
Pie chart showing the (sub)specialties requested by the 157 vacancies that were included in this study. Percentages are given with absolute numbers between parentheses. Due to the small size of the subspecialties of acute radiology, “none specified,” and pediatric radiology, these groups were combined and collectively named “other.” MSK, musculoskeletal; NHH, neuro- head and neck radiology

**Table 1:** Requested subspecialties in non-academic vs. academic vacancies

Subspecialty		Hospital	
		Non-academic (*n *= 119)	Academic (*n *= 34)
Radiology (*n *= 146)	Abdomen	21 (18%)	9 (26%)
	All-round	34 (29%)	1 (3%)
	Breast	11 (9%)	0
	Cardiothoracic	6 (5%)	5 (15%)
	Intervention	17 (14%)	5 (15%)
	MSK^1^	11 (9%)	4 (12%)
	NHH^2^	6 (5%)	5 (15%)
	Other^3^	3 (3%)	4 (12%)
Nuclear medicine (*n *= 11)	Nuclear medicine	10 (8%)	1 (3%)

The combined academic and non-academic vacancies were not included in this table

^1^*MSK*, musculoskeletal

^2^*NHN*, neuro- head and neck radiology

^3^Due to the small size of the subspecialties of acute radiology, “none specified,” and pediatric radiology, these groups were combined and collectively named “other”

**Table 2:** Distribution of requested subspecialties between permanent and locum vacancies

Subspecialty		Locum vacancies (*n *= 106)	Permanent vacancies (*n *= 51)
Radiology (*n *= 146)	Abdomen	20 (19%)	10 (20%)
	All-round	23 (22%)	12 (23%)
	Breast	9 (9%)	2 (4%)
	Cardiothoracic	9 (9%)	2 (4%)
	Intervention	11 (10%)	11 (21%)
	MSK^1^	15 (14%)	2 (4%)
	NHH^2^	8 (8%)	4 (8%)
	Other^3^	5 (5%)	3 (6%)
Nuclear medicine (*n *= 11)	Nuclear medicine	6 (4%)	5 (10%)

The combined academic and non-academic vacancies were not included in this table

^1^*MSK*, musculoskeletal

^2^*NHH*, neuro- head and neck radiology

^3^Due to the small size of the subspecialties of acute radiology, “none specified,” and pediatric radiology, these groups were combined and collectively named “other”

In the Netherlands, another factor to take into account regarding the job market is the relatively recent revision of the previously separate NM and radiology training curricula that have been combined into one integrated training in 2014 [9]. The Netherlands has been a frontrunner with this integrated training while in other European countries separate residencies for radiology and NM (with some attention to radiology) still exist. Residents who complete this integrated training can be deployed for radiology as well as NM, depending on the (sub) specialty they chose during the training [9]. In this new Dutch curriculum, a resident has to choose at least one (sub)specialty and a maximum of two subspecialties. There are seven radiology subspecialties, i.e., abdominal (ABDOM), breast, cardiothoracic (CTH), interventional (INT), musculoskeletal (MSK), neuro- head and neck (NHN), and pediatric radiology (PED), and one specialty dedicated to NM. A major change compared to previous curricula is that the residents who finish the NM pathway are called nuclear radiologists [9]. The first (nuclear) radiologists who were enrolled in this new curriculum graduated in 2019 [9]. Based on this change in the way residents are trained, some departments of radiology and NM in the Netherlands have decided to merge. This new curriculum could affect the employability and thus the job opportunities of new (nuclear) radiologists [10].

There are no recent data on vacancies for radiologists and nuclear medicine physicians in European countries such as the Netherlands, in terms of number of vacancies and the distribution of these vacancies between academic and non-academic hospitals, the most sought-after subspecialties, types of employment offered (locum or permanent employment), and requested tasks besides direct patient care (such as research, teaching, management, and ICT (information and communications technology)/AI assignments). This information is potentially useful to those who are considering or preparing to work as a medical specialist in radiology or NM in the future. Such data could also provide valuable feedback to all stakeholders who determine the contents and learning objectives of residency programs.

The purpose of this study was therefore to evaluate the current job market for medical specialists in radiology and NM in the Netherlands.

## Materials and methods

### Study design

This research used publicly available data and did not concern investigations on human subjects. Therefore, ethical review board approval and informed consent were not applicable.

### Data collection

Data collection took place between December 2020 and February 2022 via the websites of the Dutch radiology and NM societies, where all formal vacancies in these disciplines are generally listed [11, 12]. All vacancies for radiologists and nuclear medicine physicians that were posted on these websites in the aforementioned 14-month period were recorded. Vacancies for jobs outside the Netherlands and vacancies that were posted for a second time (due to not being filled in initially) were excluded. All other non-medical specialist vacancies related to radiology and NM, e.g., PhD studentships, post-doc positions, or other research-related vacancies, were excluded.

The following variables were recorded for each vacancy: the type of hospital (academic or non-academic), the type of (sub)specialty (radiology subspecialty or NM), the type of employment (fellow, locum, or permanent employment), required additional non-clinical tasks (research, teaching, management, ICT/AI, or other), the amount of full-time equivalent (FTE) for this position, and the reason for the vacancy listing.

### Data analysis

Vacancy characteristics were descriptively analyzed. Differences between non-academic and academic job postings in terms of requested subspecialisation and requests for additional research, teaching, management, or ICT/AI tasks were analyzed with a chi-square test. *p*-values < 0.05 were considered statistically significant.

Statistical analyses were performed using IBM SPSS Statistics for Windows, version 23 (IBM Corp).

## Results

### Eligible vacancies

A total of 189 vacancies were posted on the websites of the Dutch radiology and NM societies between December 2020 and February 2022. We excluded 26 vacancies for jobs abroad and 6 vacancies that were posted for a second time. The reposted vacancies were all from non-academic hospitals, concerned 4 permanent and 2 locum vacancies, and requested 3 all-round radiologists and 3 radiologists with a subspecialty (ABDOM, CTH, and INT).

### Characteristics of included vacancies

The 157 vacancies that were included concerned 119 (76%) vacancies for non-academic hospitals, 34 (22%) vacancies for academic hospitals, and 4 (2%) vacancies for a combination of academic and non-academic hospitals. There were 146 medical specialist vacancies for radiology and 11 medical specialist vacancies for NM. There were 104 locum vacancies (of which 52 for a fellowship position) and 50 permanent vacancies, while 3 vacancies did not report the type of contract. The amount of FTE per vacancy ranged from 0.2 to 1.0 with a median of 0.9 FTE. Thirty-four vacancies (22%) listed the reason for the job posting, which included the following: (temporary) replacement of a radiologist or nuclear medicine physician (*n *= 10), retirement replacement (*n *= 6), expansion of work activities or replacement (*n *= 12), or a combination of the aforementioned (*n *= 6).

The majority of vacancies did not disclose if their radiology and NM departments were fused as one department.

### Requested qualifications

The most frequently requested (sub)specialties for the 157 vacancies were as follows: all-round radiology (*n *= 35; 22%), ABDOM radiology (*n *= 30; 19%), and INT radiology (*n *= 22; 14%) (Fig. 1). Table 1 displays the distribution of subspecialty vacancies between academic and non-academic hospitals. Eight out of 11 (73%) NM vacancies specifically mentioned the search for a nuclear radiologist (i.e., residents who specialized in NM during their integrated training). Forty-eight vacancies (31%) mentioned a preference for applicants who were skilled in one or more additional non-clinical tasks. These non-clinical tasks concerned research (*n *= 39), teaching (*n *= 19), management (*n *= 11), and skills or interests in ICT/AI (*n *= 2). Note that 23 vacancies asked for more than one of the aforementioned non-clinical tasks.

### Differences between academic and non-academic vacancies

Non-academic hospitals significantly more frequently requested all-round radiologists (*n *= 34) than academic hospitals (*n *= 1) (*p *= 0.001), while the distribution of other requested subspecialties was not significantly different between non-academic and academic vacancies. Non-academic hospitals also significantly more frequently requested additional research tasks in their vacancies (*n *= 35) compared to academic hospitals (*n *= 4) (*p *= 0.011). There were non-significant trends for non-academic hospitals more frequently requesting teaching tasks in their vacancies (*n *= 18) than academic hospitals (*n *= 1) (*p *= 0.051), and for non-academic hospitals more frequently asking for management skills (*n *= 11) than academic hospitals (*n *= 0) (*p *= 0.075).

### Distribution of subspecialties between locum and permanent vacancies

All 157 vacancies mentioned the duration of the contract, of which 51 (32%) were permanent vacancies and 106 (68%) locum vacancies (Table 2). There were 52 fellowships among the locum vacancies. The most sought-after subspecialty was all-round radiology for both the permanent vacancies (*n *= 23, 22%) and locum vacancies (*n *= 12, 24%).

## Discussion

This study, which included 157 vacancies for medical specialists in radiology or NM that were published on the websites of the Dutch radiology and NM societies between December 2020 and February 2022, shows that in more than half of job postings either all-round radiologists, ABDOM radiologists, or INT radiologists were requested while the other four radiology subspecialties together with NM comprised the minority. This disbalance raises the question whether quota should be imposed to prevent unnecessary training in some subspecialties and to train sufficient residents in others. However, this speculation requires further longitudinal studies matching the qualifications of recently graduated radiologists with the demands in the job market that are probably dynamic in time. For example, further expansion of INT radiology services in the future is likely [13], and radiology departments that enlarge due to hospital mergers may aim to provide more subspecialty service. Nevertheless, the present data on subspecialty demands may already be helpful to current residents in choosing their subspecialisation.

Although the number of vacancies for nuclear medicine physicians in this study was relatively low, the majority of them specifically asked for a nuclear radiologist. This is most likely due to the versatility of a nuclear radiologist compared to a specialist trained in NM only, because the former can be deployed for both NM and radiology tasks (including on-call duties) [10]. The acceptation of and apparent preference for nuclear radiologists in the job market can be considered a potential success of the new integrated radiology and NM training and/or a reflection of the ongoing fusion of the radiology and NM departments in the Netherlands.

The far majority of vacancies in this study (approximately three-fourths) concerned jobs in non-academic hospitals. This distribution matches the number of non-academic hospitals (*n *= 74) and academic hospitals (*n *= 8) in the Netherlands [14]. Our results indicate that non-academic hospitals more frequently require all-round radiologists than academic hospitals, which seems logical because non-academic hospitals have a smaller pool of radiologists to fulfil all clinical tasks, and all-round skills increase flexibility and efficiency. Both permanent and locum vacancies in non-academic hospitals most frequently requested all-round radiologists, reflecting the high demand of all-round radiologists outside the academia. However, in the current Dutch curriculum, residents have to choose either NM or at least one radiology subspecialty, which somewhat conflicts with the time that can be evenly spent on all radiology subspecialties to become an all-rounder. This issue may be addressed in future revisions of the curriculum. On the other hand, only one out of 34 academic vacancies asked for an all-round radiologist. This indicates that, when choosing to become an all-round radiologist without subspecialisation, a career as an academic radiologist can almost be excluded in the Netherlands. Except for the all-round radiology profile, other subspecialty requests were not significantly different between non-academic and academic hospitals, indicating that radiologists with a subspecialty can find employment both non-academically and academically.

Nearly one-third of vacancies in this study asked for additional non-clinical tasks. Interestingly, non-academic hospitals also more frequently requested radiologists with research skills and tended to more frequently request teaching tasks and management skills in their vacancies than academic hospitals. Next to clinical excellence, research and teaching are important pillars in the mission statement of the non-academic top hospitals in the Netherlands [15], which is apparently reflected by the contents of the job vacancies for radiologists. It remains unclear why academic hospitals less frequently asked applicants to perform research tasks in their job postings. However, it can be postulated that performing research and teaching are considered matters of course in an academic institution. Overall, our findings highlight the necessity for all residents to acquire skills in both research and teaching, regardless of their future working place as a radiologist. The fact that management skills are more in demand by non-academic hospitals than their academic counterparts is probably related to their often more profit-driven nature and their smaller group of radiologists in which management positions frequently rotate. Importantly, although research and teaching are part of the Dutch curriculum, just like in other European and American radiology training programs, management is only trained in basics, if addressed at all [16, 17]. This issue is a potential point for improvement in these curricula. Of interest, management tasks were only requested in non-academic vacancies and not in any of the academic vacancies. This suggests that trainees who aspire a non-academic appointment should consider bolstering their management skills, while residents who pursue an academic career can perhaps better focus on research and teaching as additional non-clinical skills. In this study, only a few vacancies specifically mentioned a search for applicants who were skilled in AI or ICT tasks, while developments in this field suggest their role to rise in the near future [18]. Residents who want to best prepare for this could benefit from courses which focus on the role of AI in radiology, e.g., as provided by the ESR [19]. Finally, communication and empathy skills (so-called soft skills) were not explicitly asked for in any of the vacancies, although these aspects may also become more important in radiology practice [20]. The lack of requests for soft skills could also be due to the fact that in the Netherlands residents are trained according to CANMEDS criteria and these skills might also be tested during the application procedure [11].

Previous literature on which radiology subspecialties and skills are in demand is rather limited. Several previous studies in the USA focused on the supply and demand of radiologists on the job market. These studies reported that too many residents were trained compared to the number of available jobs in the years 2013 [21] and 2015 [1]. However, these data do not provide information on what qualifications are requested from radiologists looking for employment. In another study in which an anonymous survey was sent to Belgian radiologists (who graduated between 2013 and 2018) and to the heads of all Belgian radiology departments, it was reported that the most desired subspecialties were MSK imaging, INT radiology, and breast imaging [2]. However, these data may be less reliable because they are based on survey results and not on actual job postings. One recent study with a similar design as ours evaluated American College of Radiology (ACR) job postings in 2018, but limited their analysis to jobs labelled as MSK subspecialty [22]. In the 456 vacancies that were included in that study by Nellamattathil et al [22], approximately 19% were for a dedicated MSK radiologist, 25% sought a combination of MSK and a general skill set, and 56% were specifically for a general radiologist position. These data are somewhat in line with our findings, in that all-round radiology skills remain important. Otherwise, however, it is difficult to make a comparison with the present study, because Nellamattathil et al [22] limited their analysis to MSK job postings and did not perform any further analyses on required non-clinical skills and non-academic vs. academic vacancies.

The present study had some limitations. First, this study analyzed all vacancies posted on the radiology and NM society websites. Vacancies outside these media that were filled via non-official channels were not included. This probably means there were more vacancies available than included in this study. Second, whether or not the Dutch job market for radiologists is oversupplied or not cannot be determined in this study, because exact data regarding number of unemployed radiologists and their subspecialties vs. available vacancies were lacking. Third, the findings of this study are primarily applicable to the Netherlands. Nevertheless, some results are probably generalizable, such as the need for curricula to maintain the possibility for residents to be trained as all-round radiologists, the apparent interest of imaging departments in nuclear radiologists over nuclear medicine physicians from the previous curriculum who have no integrated radiology skills, and the need to train residents in important non-clinical skills (research and teaching, but also management), and to consider adding new elements to curricula such as ICT/AI and “soft skills” [20]. Our study also showed that a review of job vacancies over an extended period of time provides valuable information to residents and feedback to potentially improve radiology and NM residency programs. Fourth, data collection took place during the COVID pandemic which caused drastic changes in radiology practices. The periodic decline in imaging requests but also a decrease in available staff during lockdown periods might have been of influence on the job market in the Netherlands as well [23].

In conclusion, this study provided an overview of the current state of the job market for radiologists and nuclear medicine physicians in the Netherlands. All-round, ABDOM, and INT radiologists are most in demand on the job market. All-round radiologists are particularly sought after by non-academic hospitals, whereas nuclear radiologists who completed the Dutch integrated NM and radiology residency seem to be welcomed by hospitals searching for a nuclear medicine specialist. Finally, non-clinical skills (research, teaching, management, ICT/AI) are commonly requested. These data can be useful for residents and developers of training curricula.

## Abbreviations

ABDOM: Abdominal radiology
ACR: American College of Radiology
AI: Artificial intelligence
CTH: Cardiothoracic radiology
FTE: Full-time equivalent
ICT: Information and communications technology
INT: Interventional radiology
MSK: Musculoskeletal radiology
NHN: Neuro- head and neck radiology
NM: Nuclear medicine
PED: Pediatric radiology

## Declarations

### Guarantor

The scientific guarantor of this publication is Ton Velleman, University Medical Center Groningen.

### Statistics and biometry

No complex statistical methods were necessary for this paper.

### Study subjects or cohorts overlap

No know study subjects or cohorts have been previously reported.

### Methodology

- Prospective
- Observational
- Performed at one institution
